# A Method for the Immobilization of Chitosan onto Urinary Catheters

**DOI:** 10.3390/ijms232315075

**Published:** 2022-12-01

**Authors:** Alenka Vesel, Nina Recek, Rok Zaplotnik, Albert Kurinčič, Katja Kuzmič, Lidija Fras Zemljič

**Affiliations:** 1 Department of Surface Engineering, Jozef Stefan Institute, Jamova cesta 39, 1000 Ljubljana, Slovenia; 2 TIK d.o.o., Goriška cesta 5b, 5222 Kobarid, Slovenia; 3 Institute for Engineering Materials and Design, Faculty of Mechanical Engineering, University of Maribor, Smetanova 17, 2000 Maribor, Slovenia

**Keywords:** polymer, chitosan immobilization, adhesion, plasma-surface modification, biopolymers

## Abstract

A method for the immobilization of an antibacterial chitosan coating to polymeric urinary medical catheters is presented. The method comprises a two-step plasma-treatment procedure, followed by the deposition of chitosan from the water solution. In the first plasma step, the urinary catheter is treated with vacuum-ultraviolet radiation to break bonds in the polymer surface film and create dangling bonds, which are occupied by hydrogen atoms. In the second plasma step, polymeric catheters are treated with atomic oxygen to form oxygen-containing surface functional groups acting as binding sites for chitosan. The presence of oxygen functional groups also causes a transformation of the hydrophobic polymer surface to hydrophilic, thus enabling uniform wetting and improved adsorption of the chitosan coating. The wettability was measured by the sessile-drop method, while the surface composition and structure were measured by X-ray photoelectron spectroscopy and Fourier-transform infrared spectroscopy. Non-treated samples did not exhibit successful chitosan immobilization. The effect of plasma treatment on immobilization was explained by noncovalent interactions such as electrostatic interactions and hydrogen bonds.

## 1. Introduction

Chitosan is a promising natural polysaccharide material that can be used for medical and food packaging applications because of its good antibacterial, anti-inflammatory, and antioxidant properties [1,2]. Many investigations were performed to show its antibacterial properties and mode of action. More information can be found in several review papers [3,4,5,6].

The above-mentioned applications often require the deposition of a thin chitosan film on various polymer substrates. However, most polymers are hydrophobic and have low surface energy, which prevents good adhesion of a coating. Inadequate wettability causes poor contact and spreading of deposited liquid containing chitosan on the polymer surface and, consequently, poor adhesion. The polymer surface must be activated to change its surface properties. This can be obtained by various chemical treatments; however, if we want to avoid environmentally harmful chemical treatments, non-thermal plasma treatment offers a good alternative procedure. Plasma treatment enables manipulation of the surface wettability and adhesion properties of polymer materials by functionalization of their surface [7,8]. It also enables the selection of various treatment conditions and working gas, therefore offering different outputs regarding the surface properties of the treated materials. However, plasma treatment is often used as an intermediate treatment step to incorporate functional groups to which final substances are attached by using various chemical coupling agents. For example, Paslaru et al. performed corona treatment of polyethylene (PE) to covalently bond chitosan to the corona-treated surface by using 1-ethyl-3-[3-dimethylaminopropyl] carbodiimide hydrochloride (EDC crosslinking agent) and N-hydroxysuccinimide (NHS-crosslinking agent) [9]. Asadinezhad et al. performed surface dielectric-barrier discharge modification of polyvinylchloride (PVC) in the air followed by graft copolymerization with acrylic acid to form a dense polymer brush [10]. Finally, chitosan was bound to such functional surfaces. Similarly as Paslaru et al., Bahrami et al. [11] prepared chitosan-coated polyurethane films. They used nitrogen plasma treatment to attach acrylic acid first, followed by EDC chemistry (1-Ethyl-3-(3-dimethylaminopropyl)carbodiimide) to further bind chitosan to carboxylic groups for wound-dressing applications. Terpilowski et al. used nitrogen plasma for surface modification of polyetheretherketone (PEEK) [12]. In contrast to Bahrami et al., Terpilowski et al. obtained a good adhesion of chitosan which was deposited directly onto plasma-treated PEEK without any additional use of crosslinking agents. Additionally, Wiacek et al. used nitrogen/air plasma to modify PEEK surface to improve its adhesion with chitosan [13]. Besides nitrogen plasma, some authors used air plasma treatment. Carette et al. used an atmospheric plasma torch to activate polylactic acid (PLA) [14]. They performed direct dip-coating to attach chitosan to plasma-treated PLA. They concluded that stabilization of the chitosan layer was mainly induced by noncovalent interactions such as hydrogen bonding and electrostatic interactions. Demina et al. [15] also achieveddirect immobilization of chitosan onto plasma-treated substrates of polyethylene terephthalate (PET). They compared the effect of air plasma created by applying two different discharge types: direct (DC) or alternated (AC) current at 40 kHz. They proposed that chitosan was linked to plasma-treated PET films via ionic or covalent bonds. DC-plasma treatment led to a superhydrophilic surface (full spreading of a water droplet), whereas AC led to surface wettability of 17°. Small variations in oxygen and nitrogen concentrations as well as the concentrations of C-O and O=C-O groups were found for different discharge types. DC treatment also caused more significant changes in surface roughness as compared to AC. Immobilization of chitosan was observed for all plasma-treated surfaces, however, it is not clear what type of discharge was better. Suganya et al. treated polystyrene (PS) in air, argon, and oxygen plasma to immobilize chitosan onto plasma-treated PS by dip-coating to be used in food-packaging applications of fresh fruits [16]. The highest hydrophilicity of PS was obtained for oxygen plasma treatment. Oxygen plasma was found to be more effective for adhesion of chitosan than air and argon plasma. Tkavc et al. compared O_2_ and CO_2_ plasma treatment to improve adhesion of chitosan to PET surfaces [17]. The amount of nitrogen originating from chitosan coating was half lower if CO_2_ plasma was used.

The above-mentioned literature review shows that authors used different types of gases, different types of discharges, and different polymer substrates. Although these studies show that plasma can improve the adhesion of chitosan to the polymer surface, further studies are needed to optimize the procedure for different polymers. One major limitation of plasma is the diversity of chemical reactions and functional groups which are formed depending on the working gas, processing parameters (e.g., reactor configuration, discharge type, discharge power, pressure/gas flow, treatment time, etc.), and polymer type [18]. Therefore, optimal plasma-surface chemistry for one polymer is not necessarily transferable to another one. For each polymer, gas, and plasma configuration, the process needs to be optimized. It is also known that surface reactions are initiated by the formation of free radicals on the surface, followed by further binding of plasma radicals leading to the formation of different functional groups [19]. However, not all polymers have the same probability of breaking bonds and creating enough free radicals.

In the present work, we used a two-step plasma-treatment process to treat medical urinary catheters made of different polymeric materials with the aim of improving the immobilization of chitosan to the surface to obtain a chitosan-assembled hydrogel with induced hydrophilicity, resulting in better material wettability and smoothness.

The first step included hydrogen plasma treatment which was needed to create reactive-free radicals. This step was followed by the second step, where oxygen-atom functionalization was applied. We have shown that such a two-step treatment procedure can induce a more hydrophilic surface than only a one-step treatment solely in oxygen plasma.

## 2. Results and Discussion

### 2.1. Characterization of Reference Materials for Urinary Catheters

**Figure 1:**
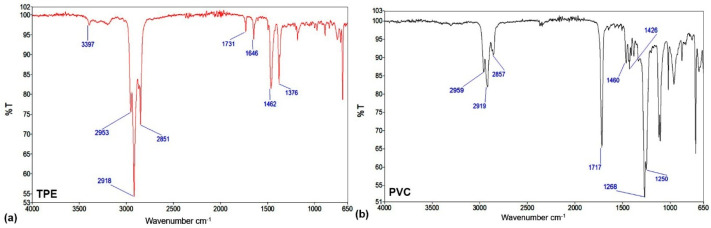
FTIR spectra of reference catheters: (**a**) TPE catheter and (**b**) PVC catheter.

For this investigation, two types of medical catheters (PVC and TPE), each one composed of several different polymers, were used. As-received catheters were first analyzed to determine the chemical composition of the surface film as probed by XPS. The results of the XPS investigation are shown in Table 1. The concentration of carbon is very high; approximately 84 and 89 atomic % for PVC and TPE catheters, respectively. The oxygen concentration is lower for TPE catheters (7.6 atomic %) as compared to PVC (11.4 atomic %). Silicon is present in minor concentrations for both catheters. TPE also contains minor concentrations of nitrogen (2.4 atomic %), whereas PVC has minor concentrations of chlorine (2.1 atomic %). A very small concentration of chlorine was found in the surface film of PVC catheters which is rather surprising. Obviously, the concentration of co-polymer polyvinyl chloride in PVC is not a dominant constituent, at least not at the surface of PVC catheter. Base catheters were also analyzed by ATR-FTIR and the spectra are shown in Figure 1. Figure 1a shows the spectrum of the base TPE catheter. There is a small vibrational band, positioned at 3397 cm^−1^, that could be characteristic of N-H or O-H stretching. There is also a strong vibrational band positioned at 2918 cm^−1^ which represents C-H stretching; a vibrational band characteristic of C=O stretching at 1731 cm^−1^; at 1646 cm^−1^ of C=C stretching; and finally, vibrational bands at 1462 cm^−1^ and 1376 cm^−1^ that are characteristic of C-H bending. Figure 1b shows the spectrum of the base PVC catheter. There is a peak at 2919 cm^−1^ corresponding to C-H stretching and one at 1717 cm^−1^ to C=O stretching. A band at approximately 1426 cm^−1^ can be attributed to C-H bending, split vibrational bands at 1268 cm^−1^ and 1250 cm^−1^ to C-O stretching from aromatic ester, and lastly a peak at 733 cm^−1^ to C-Cl stretching.

### 2.2. Effect of Plasma Treatment

#### 2.2.1. Wettability of Plasma-Treated Catheters

**Figure 2:**
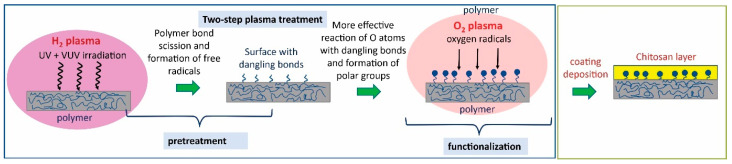
Scheme of preparation of coated catheters based on two-step plasma pretreatment followed by deposition of chitosan layers.

As-received catheters were activated by plasma treatment to improve the immobilization of chitosan. First, only oxygen plasma was used to activate the surface; however, it resulted in only a small improvement of the surface wettability. Therefore, we decided to take a different approach by using a two-step plasma treatment procedure that was recently developed by our group [20]. The novel two-step plasma treatment procedure includes the first-step treatment in hydrogen plasma, followed by the second-step treatment in oxygen plasma or its afterglow to functionalize the surface. Recently, we have shown for the polytetrafluoroethylene (PTFE) polymer that such a two-step treatment procedure enables us to obtain a superhydrophilic surface, even for chemically nonreactive polymers such as PTFE that are otherwise difficult to be modified by plasma treatment [20]. Because PVC also belongs to halogen polymers such as PTFE, we decided to apply the same procedure also for PVC and TPE catheters. Hydrogen plasma is a very intensive source of energetic UV/VUV radiation [21]. Photons have enough energy to break chemical bonds between carbon and fluorine, leading to the formation of free bonds at the surface, which is susceptible to further direct reactions with oxygen atoms. Therefore, such a two-step treatment procedure is more effective than just solely oxygen plasma treatment, and it leads to better surface functionalization and wettability. The whole concept of the preparation of plasma-treated and coated catheters is illustrated in Figure 2.

Table 2 clearly shows the benefit of using a two-step treatment procedure. If catheters were treated only with oxygen plasma, the minimum achievable water contact angles were only about 50° even when long treatment times were used (60 s). If a two-step treatment was used, the minimum water contact angles were approximately 11° and 34° for TPE and PVC catheters, respectively. It should be mentioned that these water contact angles were obtained after just 2 s of treatment with oxygen plasma. Such short treatment times are beneficial in terms of time-optimization of the treatment process because they cannot cause overtreatment or the formation of weakly bound layers of low-molecular-weight oxidized materials (LMWOM). LMWOM is detrimental to good adhesion [22,23], although it was reported in one case that LMWOM might even be incorporated into the adhesive, resulting in adhesion enhancement [24]. However, such a scenario is not very likely to occur.

**Figure 3:**
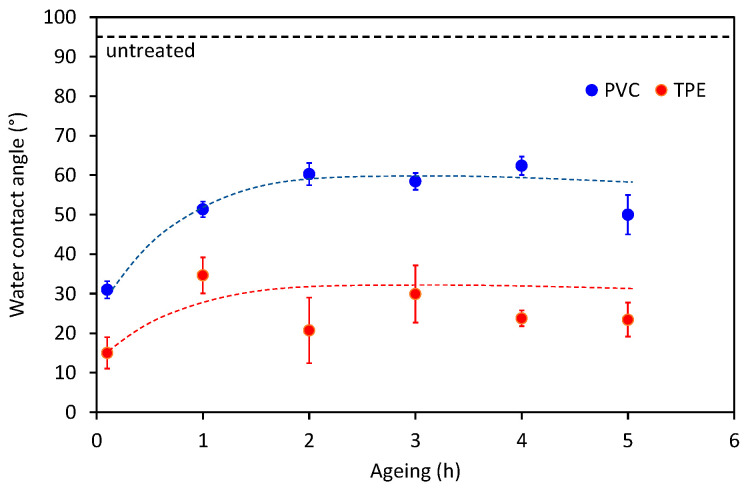
Aging of the surface of plasma-treated catheters.

Another important factor for consideration is the aging effect of the plasma-treated polymer surface because chitosan deposition was performed four hours after plasma treatment. The high surface energy is against thermodynamics, so spontaneous hydrophobic recovery occurs [25]. The hydrophobic recovery is determined by measuring the WCA at various times after the plasma treatment is accomplished. The results are shown in Figure 3. The WCA roughly doubles in the first hour after the plasma treatment, then remains fairly constant but still much lower than for non-treated samples. For the two-step plasma treatment, the water contact angle after aging was similar to the initial water contact angle of catheters treated only with oxygen plasma. Therefore, this is another benefit of the two-step plasma treatment; even if some aging occurs, the wettability is still good enough because of the lower initial (just after accomplishing plasma treatment) wettability. However, this is not the only reason for the selection of the two-step treatment as the optimal one. For the optimization of plasma treatment conditions and wettability measurements, 5-cm long samples were used, whereas entire catheters were treated when chitosan coatings were deposited. It is known that the presence of polymer materials in the discharge chamber modifies the plasma parameters [26]. When the whole catheter tube was treated in plasma, higher water contact angles were observed (see Table 2). The reason for the poorer wettability of large samples is the sink of reactive plasma species on a larger surface and because the long catheters disturb the properties of the plasma more than the smaller pieces.

**Figure 4:**
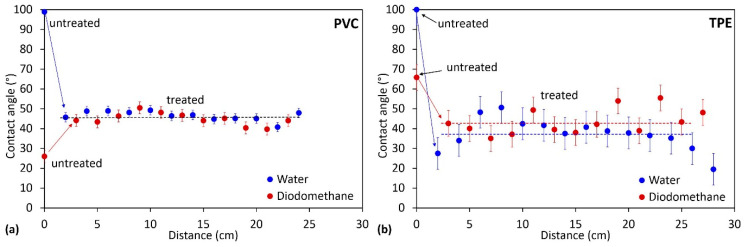
Contact angles of water and diodomethane droplets along the catheter tube: (**a**) PVC catheter and (**b**) TPE catheter. Zero on the x-axis corresponds to the end of the catheter tube which is closer to the coil (see Figure 5). Contact angles for untreated samples are added.

To check the treatment uniformity of the whole catheter surface because of the possible existence of gradients of O atoms in the plasma, we also measured the wettability along the catheter tube. The results are shown in Figure 4. The wettability was rather constant along the catheter tube, and the surface was thus uniformly treated. Table 3 and Table 4 show the average water contact angle of untreated and plasma-treated catheters, as well as the surface-free energy (SFE) together with its polar and dispersive components. For the untreated sample, SFE and the polar component of SFE were very low, whereas, after plasma treatment, they significantly increased.

#### 2.2.2. XPS Characterization of Plasma-Treated Catheters

According to the results presented above, we can conclude that two-step plasma treatment significantly improved the surface wettability of otherwise hydrophobic catheters with an initial water contact angle of approximately 100°. Further information regarding the chemical modification of surface properties of plasma-treated catheters was obtained by XPS. Table 5 shows the comparison of the surface elemental composition of the untreated and two-step plasma-treated catheters. Oxygen concentration was increased for both catheters, especially for TPE, where it was doubled. For PVC catheters, it was found that chlorine concentration was decreased after plasma treatment, which is a consequence of C—Cl bond scission by VUV radiation from hydrogen plasma, the interaction of Cl atoms with H radicals from plasma, and the desorption of HCl from the polymer. 

An increase in oxygen concentration for plasma-treated samples is a sign of the formation of new oxygen functionalities. This is also reflected in the XPS high-resolution spectra of carbon C1s. A comparison of spectra of the untreated and plasma-treated C1s peaks of catheters is shown in Figure 6a,c for TPE and PVC catheters, respectively. An increase in the intensity of the tail on the high-binding energy side of the C1s spectra is observed because of the formation of new functional groups. In Figure 6b,d are shown fitted spectra of plasma-treated samples showing the increase of the content of C-O, C=O, and O=C-O functional groups as a consequence of the plasma treatment procedure.

To summarize, as shown by wettability and XPS measurements above, plasma treatment has two beneficial effects: (1) it increases the surface wettability and uniformity of spreading the liquid, and (2) it causes the formation of new functionalities that may act as binding sites for the coating immobilization.

### 2.3. Analysis of Plasma-Treated Catheters with the Chitosan Coating

#### 2.3.1. XPS Characterization of Plasma-Treated Catheters

Two-step plasma-treated samples were coated with one or two layers of chitosan. Furthermore, two different concentrations of chitosan were used: 2% and 2.5% wt. macromolecular chitosan solution. XPS-elemental chemical composition of the coated catheters is shown in Table 6 and Table 7 for TPE and PVC catheters, respectively. We can observe a significant increase in oxygen and nitrogen concentrations for all catheters which were activated by plasma treatment, which proves the presence of chitosan coating on the surface. The chemical structure of chitosan has many OH and NH_2_ groups as well as C-O-C groups. This is a reason for the increased concentration of oxygen and nitrogen on the surface of catheters, proving the successful immobilization of chitosan. For untreated samples soaked in the chitosan solution, the surface chemical composition was similar to base catheters shown in Table 1, meaning that there was only minor attachment of chitosan, because the amount of nitrogen is low. The hydrophobicity of the untreated catheters was too high, and it prevented good wetting of the surface and contact with the chitosan solution. Results in Table 6 and Table 7 thus clearly prove that catheters must be activated by plasma if we want to immobilize chitosan to their surface and achieve its effect, i.e., better smoothness and/or antimicrobial activity. 

Within the experimental error of XPS methods, there were no statistically significant differences when comparing 2% or 2.5% wt. macromolecular chitosan solution for plasma-treated PVC. In contrast, for TPE, 2.5% wt. solution of chitosan led to higher oxygen and nitrogen content on the surface. When comparing one or two layers, there was no difference for TPE catheters, meaning that one layer is already thick enough and/or spread uniformly on the surface area probed by XPS. In contrast, for PVC, two layers of chitosan gave better results regarding oxygen and nitrogen concentrations, as will also be shown later by showing high-resolution C1s spectra.

The results shown in Table 6 and Table 7 are further supported by the high-resolution carbon spectra of the coated samples presented in Figure 7 and Figure 8. Figure 7a,b and Figure 8a,b show a comparison of C1s peaks of untreated TPE and PVC catheters with one or two coatings with 2% and 2.5% wt. macromolecular chitosan solution. There is no noteworthy difference, meaning that if the catheter was not activated in plasma, chitosan was not attached to the surface. On the contrary, Figure 7c,d and Figure 8c,d clearly show a remarkable difference when comparing plasma-treated and coated samples with the uncoated ones. Significant peaks observed at 286.5 eV and 288 eV correspond to C-OH/C-NH_2_ and O-C-O groups of chitosan, respectively. In Figure 8c,d, the peaks at 286.5 eV and 288 eV are more intense if two layers of the coating were present on the PVC catheter, as already mentioned above. Apparently, this is not the case for TPE catheters (Figure 7c,d).

Figure 7e and Figure 8e show the nitrogen N1s spectra of the coated samples. The source of nitrogen is amino groups from chitosan coating. By detailed observation of nitrogen peaks, more information on the binding mechanism of chitosan can be obtained. Therefore, in Figure 9, selected nitrogen peaks are shown for both plasma-treated catheters with a two-layer coating. N1s spectra were fitted with two components—the main one at 399.3 eV corresponding to amine and amide groups, and the smaller one at 401.2 eV corresponding to protonated nitrogen. The appearance of the latter peak may be explained by the formation of electrostatic interactions or hydrogen bonding between plasma-induced oxygen functionalities and chitosan molecules. In particular, carboxyl functional groups on the catheter surface may interact with ammonium functional groups of chitosan. There may also be hydrogen bonding between OH groups and van der Waals forces (orientation ones). The same observation was also reported by Carette et al. [14]. Such interactions are well-known to appear in hydrogels [27,28]. The chitosan is thus mainly immobilized by noncovalent interactions, as illustrated in Figure 10. One possible mechanism of immobilization may also be covalent binding via the formation of an amide group; however, amide and amines cannot be resolved in N1s spectra.

#### 2.3.2. FTIR Characterization of Plasma-Treated Catheters

FTIR spectra additionally confirm the XPS results presented above. FTIR spectra of coated TPE and PVC catheters, compared to reference materials (uncoated TPE and PVC catheters as well as dry chitosan powder), are shown in Figure 11 and Figure 12. It is clear that two-step plasma treatment significantly contributed to improving the attachment of chitosan to the surface of catheters (Figure 11b,d and Figure 12b,d). The band at 3277 cm^−1^ characteristic of -OH and -NH_2_ stretching vibrations; vibrational bands at 1639 cm^−1^ and 1547 cm^−1^ that correspond to CONH_2_ and -NH_2_ groups, respectively; and a peak at 1025 cm^−1^ which is attributed to C-O-C bonds, together confirm the presence of chitosan on catheters and thus an efficient attachment. In contrast, the spectra of coated catheters that were not pretreated in plasma (Figure 11a and Figure 12a) more or less completely overlap with the spectra of the reference catheters. No or very little chitosan was found on the surface of the coated catheters; there was slight absorbance at wavenumbers of chitosan-characteristic vibrational bands at approximately 1587 cm^−1^ and also in the fingerprint peak at approximately 1060 cm^−1^ (Figure 11a), or no apparent absorbance at chitosan characteristic vibrational bands at all (Figure 12a). 

However, Figure 11c and Figure 12c, where two layers of the chitosan coating were applied, show higher absorbance in the characteristic vibrational bands mentioned before. This appears to be the case for both lower and higher concentrations of chitosan used for the coating. Contrarily, there is no significant difference between the spectra of the two-step plasma-treated catheters coated with 2.0% wt. or 2.5% wt. macromolecular chitosan solution.

#### 2.3.3. Wettability of the Coated Catheters

Catheter wettability is a critical application parameter. The surface of commercially available catheters should be highly hydrophilic to allow smooth insertion and withdrawal of the catheter. If the catheter surface has little or no lubricity, this will result in microtrauma or tiny cuts in the urethral tissue. Urethral trauma can lead to hematuria (blood in the urine), pain, further infection, and/or discomfort [23]. Therefore, it is important to improve hydrophilicity of the catheters. One of the very attractive approaches is the development of highly hydrophilic and lubricious coatings. However, it is necessary to ensure that such highly hydrophilic coating films are firmly fixed on the surface of catheters with binding force, which can reduce surface friction with human tissue during the use of interventional catheters, improve patient comfort, and effectively reduce the occurrence of infection. Therefore, monitoring the water contact angle of the functionalized catheter surface is of great value to estimate their wettability and hydrophilicity. The water contact angles of the catheters coated with the two macromolecular chitosan solutions (2% and 2.5% wt.) are shown in Table 8 and Table 9 for TPE and PVC catheters, respectively. Especially when the coatings are inserted into sterile water or sterile saline, the hydrophilic coating should be activated through swelling and ready for easy and smooth insertion without the risk of urethral injury. Therefore, the water contact angle of reference and chitosan-coated catheters were measured both in the dry state (marked as WCA_dry_) and after prior activation with sterile saline (marked as WCA_wet_).

The reference catheters themselves are hydrophobic, and after plasma activation, the water contact angle for both materials dropped below 50° (see Table 3 and Table 4). The water contact angle on the surface of the compact chitosan tablet (as reference for chitosan) was also measured, and shown to be 84.5° with a total surface free energy according to OWRK model SFE = 32.6 ± 3.7 mN/m, where the dispersive part was 28.8 ± 3.3, and the polar part 3.8 ± 1.6 mN/m. This means that although chitosan is moderately hydrophilic, it still has a fairly high water contact angle with a high proportion of the dispersive part and a very low proportion of the accessible polar part. Obviously, carbohydrate chains and impurities detected in commercial chitosan as fatty acids and alkanes may have an influence on such a high dispersive fraction in this polymer [29].

After applying chitosan coatings to untreated catheters and to plasma-treated catheters, the water contact angles for dry catheters WCA_dry_ were only insignificantly changed compared to the reference catheters, so wettability did not change significantly. For TPE-coated catheters (plasma-treated and untreated), a slight decrease in water contact angle between 1% and 21% was generally observed compared to the reference. TPE catheters, whether plasma-activated or not, coated with a layer of 2% macromolecular solution exhibited a hydrophobic character with a water contact angle greater than 100°. In particular, for the catheters not treated with plasma, this may be a consequence of the absence of chitosan on the catheter surface, thus showing similar wettability as the reference catheter. A two-layer coating caused the water contact angle of catheters to decrease to approximately 82° (which is similar to the wettability of the chitosan reference), regardless of whether they had been plasma treated or not. In the case of a 2.5-macromolecular solution of chitosan as a single-layer coating, a hydrophobic character can be seen, while in the case of a two-layer coating, a decrease in the water contact angle can again be observed. This can be explained with the help of the XPS results. It has been shown that with two-layer coatings, a much larger amount of chitosan accumulates on the surface, forming a thicker structure capable of swelling and producing a hydrogel effect. Similar results can be observed for two-step plasma-treated TPE, which was subsequently coated with a macromolecular chitosan solution (2% and 2.5% wt.) in the wet phase. Apparently, plasma activation has resulted in such an amount (see XPS) and conformation of chitosan that a super hydrophilic surface is obtained by wetting with sterile saline. 

PVC catheters in the WCA_dry_ form show a small increase of the water contact angle for most samples with chitosan coatings that have not been treated with plasma. Here, the application of plasma treatment shows a more consistent effect. For all samples pretreated with a two-step plasma treatment and applying coatings of chitosan (either 2% or 2.5% wt. of the macromolecular solution), the water contact angle generally decreased by more than 10% (exception: two-step plasma treated PVC + 2.5% chitosan (one layer)). A hydrophilic surface was obtained for all samples which were pretreated in plasma and then coated with chitosan, with the exception of the two-step plasma-treated PVC + chitosan 2.5% (one layer), where the water contact angle was 91.7°. However, in all cases, the water contact angles for WCA_dry_ samples were still high and did not significantly improve smoothness.

In general, untreated catheters are thought to have little or no affinity for binding chitosan. The latter depends specifically on the chitosan concentration and layers. When chitosan is bound to the surface, it obviously happens in such a way that the polar groups are not as present on the surface and the roughness is not as pronounced due to very poor chitosan binding. It is known that these two factors—polar groups and increased roughness—improve wettability by reducing the water contact angle [24]. It seems that the dispersed fraction dominates in non-plasma-activated samples, so it is expected that chitosan adheres to the material surface of TPE and PVC reference catheters by physical (van der Waals forces) and hydrophobic interactions, and hydrophobic chains are mainly present on the surface.

Plasma treatment increases the polar fraction in both materials, PVC and TPE, which is about 1/3 of the total fraction in PVC and 41% in TPE. In this case, Coulomb electrostatic interactions and hydrogen bonding may occur, which should generally invert the polarity but, on the other hand, may also reduce the availability of free OH groups and amino and carboxylic acids, so that the polarity and wettability do not increase significantly in the dry stage either. After wetting the coatings with sterile saline, the water contact angle became zero in all cases. After application of the coating to the surface of two-step plasma-treated catheters, the immobilization of chitosan is better (as shown by XPS). Thus, chitosan can form homogeneous and thick polyelectrolyte films with gelling properties (hydrogel effects) and a structure that swells to such an extent that it significantly affects wettability and becomes super hydrophilic. It has already been shown that chitosan is able to successfully form hydrogels that can maintain a moist environment and mimic human soft tissue [25]. The homogeneity is also the result of a possible electrostatic interaction between plasma-activated catheters and chitosan, leading to monolayer adsorption and a more controlled distribution of coatings on the surface. The fact that all untreated plasma samples coated with chitosan in the wet state have a similar water contact angle as samples in the dry state and the reference material clearly proves the fact that, in some cases, there is no or very little chitosan on the surface, as clearly discussed in the XPS section.

## 3. Materials and Methods

### 3.1. Materials and Chemical Reagents

Two types of urinary medical catheters were supplied by TIK d.o.o. (Kobarid, Slovenia). They were made of Meliflex XP polyolefin-based compounds for medical applications provided by Melitek (Nørre Alslev, Denmark). The first one was based on thermoplastic elastomer (afterward marked as TPE catheter), and the other one was based on polyvinyl chloride polymer material (afterward marked as PVC catheter). The catheters were tubular with a length of 40 cm and an outer diameter of 3.5 mm.

Chitosan (low molecular weight; Sigma-Aldrich Chemie GmbH, Taufkirchen, Germany) and Milli-Q/ultrapure water (resistivity of 18.2 MΩ cm at 25 °C) were used to prepare the solution by using the Milli-Q system (Millipore Corporation, Danvers, MA, USA). The pH of the solutions was adjusted by adding acetic acid. 

### 3.2. Preparation of Coated Urinary Catheters

#### 3.2.1. Plasma Treatment of Catheters

**Figure 5:**
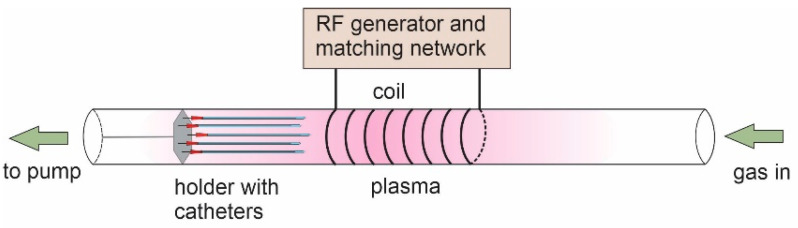
Experimental set-up.

**Figure 6:**
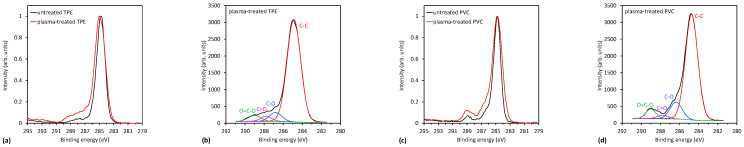
(**a**,**c**) Comparison of C1s peaks of untreated and two-step plasma-treated TPE and PVC catheters and (**b**,**d**) best fitting of C1s peaks with oxygen-containing functional groups for plasma-treated TPE and PVC catheters.

**Figure 7:**
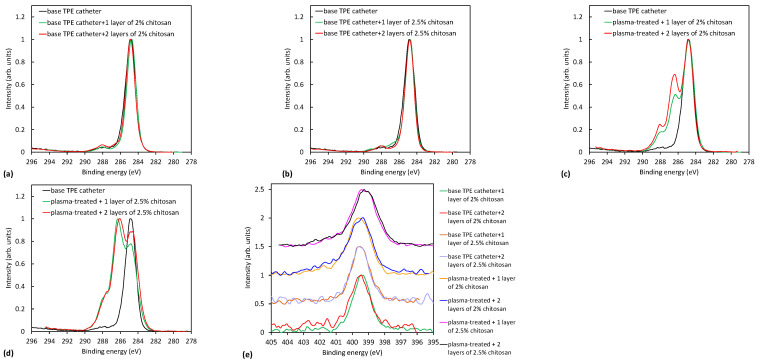
Comparison of XPS spectra of untreated and uncoated TPE catheters with coated catheters: (**a**) Soaking in 2% chitosan solution without plasma treatment; (**b**) Soaking in 2.5% wt. macromolecular chitosan solution without plasma treatment; (**c**) 2% chitosan coating with two-step plasma treatment; (**d**) 2.5% wt. macromolecular chitosan coating with two-step plasma treatment; and (**e**) comparison of nitrogen spectra for all coated TPE catheters.

**Figure 8:**
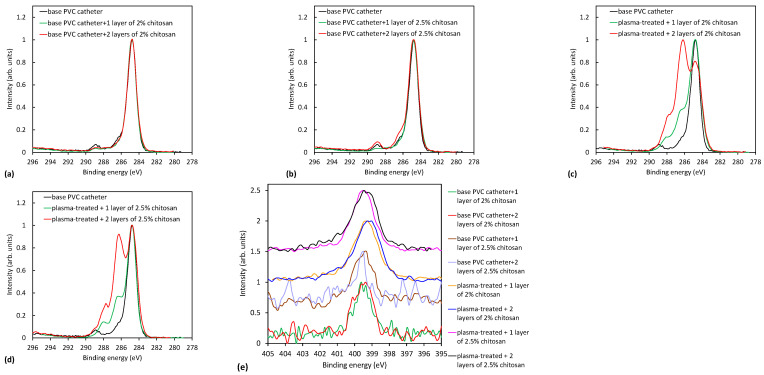
Comparison of XPS spectra of untreated and uncoated PVC catheters with coated catheters: (**a**) soaking in 2% chitosan solution without plasma treatment; (**b**) soaking in 2.5% wt. macromolecular chitosan solution without plasma treatment; (**c**) 2% chitosan coating with two-step plasma treatment; (**d**) 2.5% wt. chitosan coating with two-step plasma treatment; and (**e**) comparison of nitrogen spectra for all coated PVC catheters.

**Figure 9:**
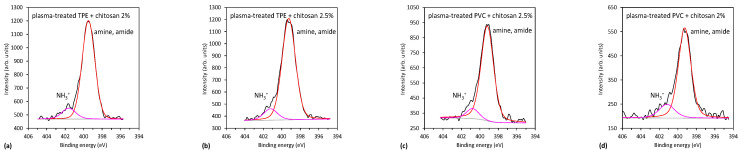
XPS nitrogen peaks of two-step plasma-treated catheters with chitosan coating (two layers): (**a**) TPE with 2% chitosan coating, (**b**) TPE with 2.5% chitosan coating, (**c**) PVC with 2% chitosan coating and (**d**) PVC with 2.5% chitosan coating.

**Figure 10:**
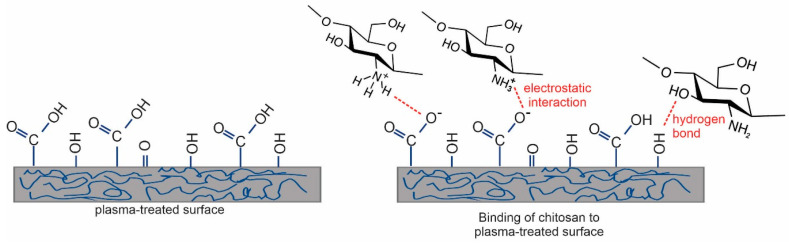
Different types of interaction of chitosan with the plasma-treated surface.

**Figure 11:**
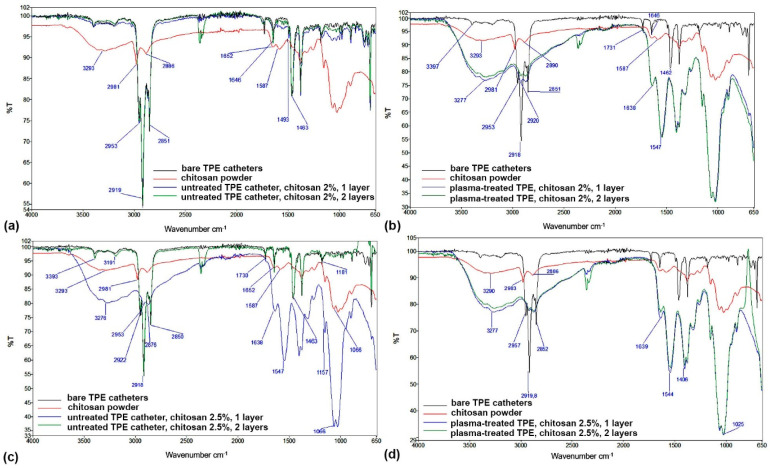
FTIR spectra of TPE catheters with chitosan coatings: (**a**) untreated catheter with one or two layers of the 2% chitosan coating, (**b**) plasma-treated catheter with one or two layers of the 2% chitosan coating, (**c**) untreated catheter with one or two layers of the 2.5% chitosan coating and (**d**) plasma-treated catheter with one or two layers of the 2.5% chitosan coating. In all subfigures, reference spectra of the bare TPE catheter and chitosan powder were added.

**Figure 12:**
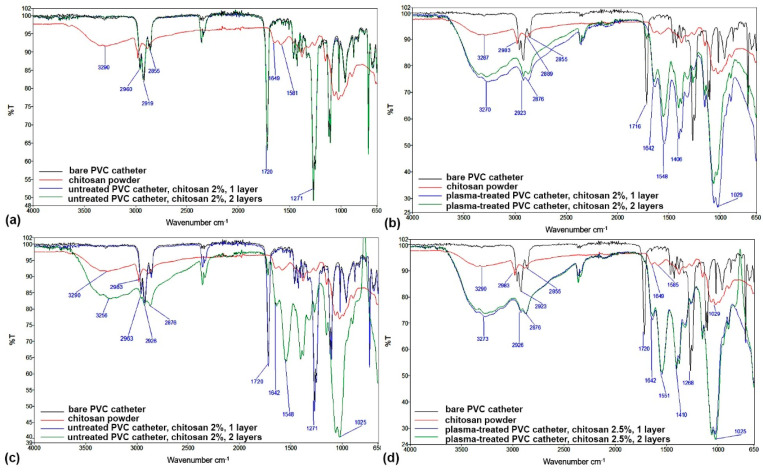
FTIR spectra of PVC catheters with chitosan coatings: (**a**) untreated catheter with one or two layers of the 2% chitosan coating, (**b**) plasma-treated catheter with one or two layers of the 2% chitosan coating, (**c**) untreated catheter with one or two layers of the 2.5% chitosan coating and (**d**) plasma-treated catheter with one or two layers of the 2.5% chitosan coating. In all subfigures, reference spectra of the bare PVC catheter and chitosan powder were added.

Catheters were treated in a custom-made large-scale radiofrequency (RF) plasma reactor to activate their surface prior to coating deposition. The plasma reactor is shown schematically in Figure 5. The discharge chamber was a 2-m long glass tube with an inner diameter of 18 cm. The reactor was pumped with a two-stage rotary-vacuum pump with a pumping speed of 80 m^3^ h^−1^. The pressure was measured with calibrated absolute gauges. The base pressure in the system was approximately 1 Pa. Gases were introduced through gas-flow controllers calibrated for hydrogen or oxygen. Plasma was sustained by the RF generator operating at a frequency of 27.12 MHz and adjustable output power up to approximately 8 kW. A copper coil was connected to the RF generator via a matching network, as illustrated in Figure 5.

Catheters were mounted to a special holder and placed inside the tube, as shown in Figure 5. Catheters were first treated with hydrogen plasma for 30 s to make dangling bonds at the surface. After hydrogen plasma treatment, they were further treated with oxygen plasma for 2 s to functionalize the surface with polar functional groups. These treatment times were previously determined as the most optimal. Hydrogen plasma was sustained at a flow rate of 100 sccm (20 Pa), and a forward RF power of 2200 W. Oxygen plasma was sustained at 300 sccm (45 Pa) using the same power as hydrogen plasma. For comparison, in another set of experiments, catheters were treated only with oxygen plasma to prove the beneficial effect of hydrogen pretreatment on surface wettability. After plasma treatment, the catheters were coated with a layer of chitosan.

#### 3.2.2. Deposition of Chitosan Coating

**Figure 13:**
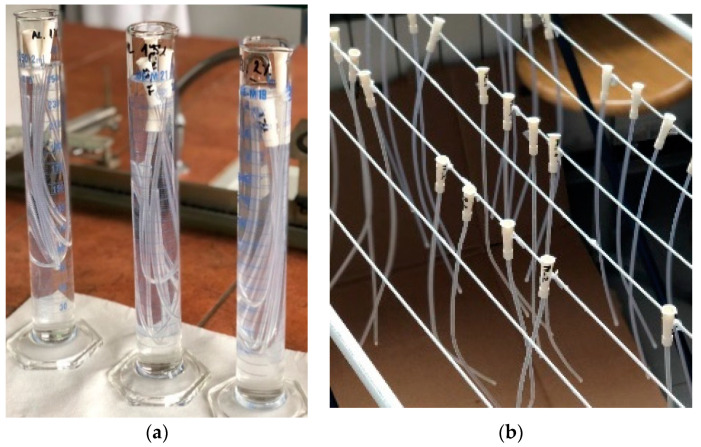
(**a**) Soaking of catheters in coating solution and (**b**) drying of catheters.

2% wt. and 2.5% wt. macromolecular chitosan solutions were prepared, respectively, with an appropriate weight of chitosan in a given volume of acidified distilled water (pH = 3.6, adjusted with concentrated acetic acid). Catheters were soaked in these chitosan solutions (Figure 13a) for 20 min. They were drained for approximately 30 s and air-dried (Figure 13b) by hanging them on a drying rack and leaving them to dry for 24 h. After 24 h, the deposition was repeated to make a two-layer coating.

### 3.3. Surface Characterization

#### 3.3.1. Chemical Composition by XPS and FTIR

The chemical composition of as-received catheters, plasma-treated catheters, and catheters with the chitosan coating before and after desorption experiments was analyzed by X-ray photoelectron spectroscopy (XPS) and Fourier-transform infrared spectroscopy (FTIR). 

XPS characterization was performed with the instrument model TFA XPS (from Physical Electronics, Munich, Germany). The catheters were irradiated with monochromatic Al Kα_1,2_ radiation with a photon energy of 1486.6 eV. The diameter of an analysis area was 400 μm. Photoelectron spectra were measured at an electron take-off angle of 45°. Survey spectra were acquired at a pass energy of 187 eV using an energy step of 0.4 eV. High-resolution C1s and N1s spectra were measured at a pass energy of 29.35 eV using an energy step of 0.125 eV. An additional electron gun was used for compensation of the surface charge. The spectra were referenced against the C1s peak at 284.8 eV. Measured spectra were analyzed using MultiPak v8.1c software (Physical Electronics, Munich, Germany), which was supplied with the spectrometer. Shirley-type background subtraction was used. 

FTIR spectra were acquired with the spectrometer ATR FTIR Perkin Elmer Spectrum GX (Perkin Elmer FTIR supplied by Omega, Ljubljana Slovenia). The ATR accessory (supplied by Specac Ltd., Orpington, Kent, UK) contained a diamond crystal. Spectra were recorded at ambient temperature in wavenumber intervals between 4000 and 650 cm^−1^; 16 scans per sample with a resolution of 4 cm^−1^. Catheters were cut into smaller samples, approximately 1-cm long, then cut in half and analyzed on the outer surface.

#### 3.3.2. Surface Wettability

The surface wettability of catheters before and after plasma treatment was monitored by measuring liquid contact angles using the Drop Shape Analyser DSA 100 and software Advance (version 1.10.1.09301) from Krüss GmbH (Hamburg, Germany). A static contact angle was measured using a sessile-drop method. The volume of a droplet was set to 1 µL. MilliQ water and diiodomethane were used for droplets to determine the surface-free energy. The water contact angle (WCA) was measured to determine the wettability. Several measurements were recorded along the catheter to check the homogeneity of the plasma effect. Water and diiodomethane liquids were used which were placed alternately every 5 mm along the catheter. This also enabled determination of surface-free energy using the OWRK method (Owens, Wendt, Rabel and Kaelble model). The surface tensions of liquids (γ_L_), i.e., water and diiodomethane are shown in Table 10.

The wettability of the chitosan-coated catheters was measured with the optical contact angle meter OCA 35 and the SCA 20 software (version 4.1.12) (DataPhysics Instruments, Filderstadt, Germany). To measure the contact angle of chitosan, a chitosan tablet was prepared by adding 15 g of chitosan powder to the pill dispenser using a Perkin–Elmer hydraulic press with pressing conditions of 1 min. In addition to water, the following liquids were used to determine the contact angle on the tablet surface: ethanol, ethylene glycol, diiodomethane and glycerol, with the total surface tension (γ_L_^tot^) and their polar (γ_L_^P^) and dispersive (γ_L_^D^) components as shown in Table 1. Three repetitions of measurements were performed. These probe fluids were used to determine the surface-free energy of chitosan using the OWRK method as described in [30,31].

## 4. Conclusions

Catheters made from commercial polymer blends containing either thermoplastic elastomer or polyvinyl chloride were treated with non-equilibrium gaseous plasma to obtain an adequate surface finish for appropriate immobilization of a chitosan coating. A cylindrical plasma reactor with an inner diameter of 18 cm and length of 200 cm was suitable for mounting several catheters of =40-cm length. The catheters were treated with weakly ionized oxygen plasma for various periods, and the wettability was measured by the sessile-drop method just after the treatment. The wettability of both types of catheters was increased moderately by the sole oxygen plasma treatment. The water contact angle was originally approximately 100° and decreased to approximately 75° after 2 s of plasma treatment. It kept decreasing with increasing treatment time before stabilizing at approximately 50° after a minute of treatment with oxygen plasma. Such modest wettability was explained by the peculiarities of the polymer blends, which did not allow for appropriate functionalization with polar oxygen-containing groups. Much better wettability was obtained by subsequent exposure to hydrogen and oxygen plasmas. The hydrogen plasma is a rich source of VUV radiation with photon energy exceeding any binding energy in the polymer material, so the absorption of VUV photons within the surface film caused bond scission. The dangling bonds were occupied with hydrogen atoms to form a polyolefin-like structure typical for plasma polymers. Such a structure was then functionalized with polar groups by a 2 s exposure to oxygen plasma. Under the optimal conditions (i.e., hydrogen plasma treatment time of 30 s followed by 2 s oxygen plasma), the WCA was 34° and 11° for PVC and TFE samples, respectively. The polar component of the surface-free energy increased from a very low value 0.03 mN/m, typical for hydrophobic polymers, to 21 and 26 mN/m, respectively. Such a large polar component enabled an optimal immobilization of a chitosan film on the plasma-treated catheters. While only minor amounts of chitosan were detected on the untreated catheters after incubating in a water solution of chitosan, the plasma-treated catheters exhibited a rather uniform coating, which was deduced from the examination of XPS and FTIR results. Based on these results, a model of interaction between plasma-treated catheters and chitosan films was proposed. Furthermore, water contact angle measurements proved that plasma activation resulted in such a successful amount and conformation of chitosan on the surface that it can form a superhydrophilic surface and thus a large lubricity potential after wetting with sterile saline. The methods are applicable to urinary catheters where a layer of water-soaked chitosan replaced the classical lubricants.

## Figures and Tables

**Table 1:** XPS surface composition of as-received urinary medical catheters (atomic %).

Type of Catheter	C	N	O	Si	Cl
Base TPE catheter	88.7	2.4	7.6	1.4	/
Base PVC catheter	84.1	/	11.4	2.4	2.1

**Table 2:** Wettability of plasma-treated catheters.

Catheter Material	Treatment Condition	Water Contact Angle (°) When Treating Small Samples	Water Contact Angle (°) When Treating the Whole Catheter
PVC	Untreated	96°	/
TPE		103°	
PVC	2 s O_2_	74°	/
TPE		77°	
PVC	30 s O_2_	60°	/
TPE		56°	
PVC	60 s O_2_	52°	/
TPE		48°	
PVC	30 s H_2_ + 2 s O_2_	34°	46°
TPE		11°	37°

**Table 3:** Wettability, surface free energy (SFE), the disperse and polar components of SFE for PVC catheter.

PVC Catheter	Untreated	Two-Step Plasma Treatment
Water contact angle:	96.1° ± 1.7°	46.3° ± 3.3°
Surface free energy:	46.7 mN/m	59.0 mN/m
Dispersive component:	45.6 mN/m	37.1 mN/m
Polar component:	0.1 mN/m	21.4 mN/m

**Table 4:** Wettability, surface-free energy (SFE), the disperse and polar components of SFE for TPE catheter.

TPE Catheter	Untreated	Two-Step Plasma Treatment
Water contact angle:	103.1° ± 1.5°	37.2° ± 8.0°
Surface free energy:	25.1 mN/m	64.2 mN/m
Dispersive component:	25.3 mN/m	37.9 mN/m
Polar component:	1.7 mN/m	26.4 mN/m

**Table 5:** XPS composition of plasma-treated catheters (in atomic %).

Sample	C	N	O	Si	Cl	O/C
Untreated TPE	88.7	2.4	7.6	1.4		0.09
Two-step plasma-treated TPE	83.0	1.9	15.0	0.1		0.18

**Table 6:** XPS composition of TPE catheters with the coating (in atomic %).

Treatment Condition for TPE Catheters	C	N	O	Si	S
untreated TPE + chitosan 2% (one layer)	86.7	2.9	9.4	1.1	
two-step plasma-treated TPE + chitosan 2% (one layer)	71.8	5.1	20.9	2.0	0.1
untreated TPE + chitosan 2% (two layers)	86.1	2.6	10.5	0.8	
two-step plasma-treated TPE + chitosan 2% (two layers)	71.3	4.7	22.6	1.2	0.2
untreated TPE + chitosan 2.5% (one layer)	84.7	3.5	10.7	1.1	
two-step plasma-treated TPE + chitosan 2.5% (one layer)	65.8	6.3	27.8	0	0.1
untreated TPE + chitosan 2.5% (two layers)	86.8	2.5	10.2	0.4	
two-step plasma-treated TPE + chitosan 2.5% (two layers)	66.9	5.6	26.9	0.4	0.1

**Table 7:** XPS composition of PVC catheters with the coating (in atomic %).

Treatment Condition for PVC Catheters	C	N	O	Si	Cl
untreated PVC + chitosan 2% (one layer)	85.2	3.1	8.9	1.6	1.2
two-step plasma-treated PVC + chitosan 2% (one layer)	76.3	4.7	17.5	1.5	
untreated PVC + chitosan 2% (two layers)	86.6	1.6	8.6	1.9	1.3
two-step plasma-treated PVC + chitosan 2% (two layers)	63.8	5.9	28.9	1.4	
untreated PVC + chitosan 2.5% (one layer)	85.3	2.3	9.0	1.9	1.5
two-step plasma-treated PVC + chitosan 2.5% (one layer)	76.5	4.5	17.6	1.4	
untreated PVC + chitosan 2.5% (two layers)	82.8	1.7	12.0	1.5	2.0
two-step plasma-treated PVC + chitosan 2.5% (two layers)	67.6	5.0	26.8	0.5	

**Table 8:** Water contact angle (WCA) of TPE catheters with the coatings.

Treatment Condition for TPE Catheters	WCA_dry_ (°)	WCA_wet_ (°)
Reference TPE	103.1	107.3
untreated TPE + chitosan 2% (one layer)	100.3	75.7
two-step plasma-treated TPE + chitosan 2% (one layer)	107.3	0
untreated TPE + chitosan 2% (two layers)	82.8	62.7
two-step plasma-treated TPE + chitosan 2% (two layers)	82.3	0
untreated TPE + chitosan 2.5% (one layer)	100.9	82.0
two-step plasma-treated TPE + chitosan 2.5% (one layer)	105.9	0
untreated TPE + chitosan 2.5% (two layers)	96.4	83.6
two-step plasma-treated TPE + chitosan 2.5% (two layers)	90.2	0

**Table 9:** Water contact angle (WCA) of PVC catheters with the coatings.

Treatment Condition for PVC Catheters	WCA_dry_ (°)	WCA_wet_ (°)
Reference PVC	96.1	104.6
untreated PVC + chitosan 2% (one layer)	102.3	97.7
two-step plasma-treated PVC + chitosan 2% (one layer)	77.1	0
untreated PVC + chitosan 2% (two layers)	99.7	89.3
two-step plasma-treated PVC + chitosan 2% (two layers)	81.2	0
untreated PVC + chitosan 2.5% (one layer)	98.6	95.7
two-step plasma-treated PVC + chitosan 2.5% (one layer)	91.7	0
untreated PVC + chitosan 2.5% (two layers)	96.7	95.0
two-step plasma-treated PVC + chitosan 2.5% (two layers)	78.7	0

**Table 10:** Total surface tension of the liquids (γ_L_^tot^) and their polar (γ_L_^P^) and dispersive (γ_L_^D^) components.

Liquid	γ_L_^tot^ (mN/m)	γ_L_^D^ (mN/m)	γ_L_^P^ (mN/m)
water	72.8	21.8	51
ethanol	22.4	18.8	3.6
ethylene glycol	48	29	19
diiodomethane	50.8	50.8	0
glycerol	64	34	30

## Data Availability

Not applicable.
